# Altered DNA methylation in kidney disease: useful markers and therapeutic targets

**DOI:** 10.1007/s10157-022-02181-5

**Published:** 2022-01-13

**Authors:** Kaori Hayashi

**Affiliations:** Division of Nephrology, Endocrinology and Metabolism, Department of Internal Medicine, Keio University School of Medicine, 35 Shinanomachi, Shinjuku-ku, Tokyo, 160-8582 Japan

**Keywords:** Kidney, Epigenetic changes, DNA methylation, DNA damage repair

## Abstract

Recent studies have demonstrated the association of altered epigenomes with lifestyle-related diseases. Epigenetic regulation promotes biological plasticity in response to environmental changes, and such plasticity may cause a ‘memory effect’, a sustained effect of transient treatment or an insult in the course of lifestyle-related diseases. We investigated the significance of epigenetic changes in several genes required for renal integrity, including the nephrin gene in podocytes, and the sustained anti-proteinuric effect, focusing on the transcription factor Krüppel-like factor 4 (KLF4). We further reported the role of the DNA repair factor lysine-acetyl transferase 5 (KAT5), which acts coordinately with KLF4, in podocyte injury caused by a hyperglycemic state through the acceleration of DNA damage and epigenetic alteration. In contrast, KAT5 in proximal tubular cells prevents acute kidney injury via glomerular filtration regulation by an epigenetic mechanism as well as promotion of DNA repair, indicating the cell type-specific action and roles of DNA repair factors. This review summarizes epigenetic alterations in kidney diseases, especially DNA methylation, and their utility as markers and potential therapeutic targets. Focusing on transcription factors or DNA damage repair factors associated with epigenetic changes may be meaningful due to their cell-specific expression or action. We believe that a better understanding of epigenetic alterations in the kidney will lead to the development of a novel strategy for chronic kidney disease (CKD) treatment.

## Introduction

Epigenetic changes represent heritable changes in gene expression that do not involve changes in the underlying DNA sequence, namely, a change in phenotype without a change in genotype. Epigenetic mechanisms include DNA methylation, histone modifications and RNA-based regulation, such as noncoding RNAs, long noncoding RNAs and microRNAs [1].

Although epigenetic changes have been investigated mainly in aging and tumorigenesis, recent studies have demonstrated an important role of epigenetic changes in lifestyle-related diseases. Epigenetic regulation promotes biological plasticity in response to environmental changes and enables transgenerational transmission of the responses. Such plasticity may cause a ‘memory effect’, a sustained effect of a transient treatment or an insult in disease courses. Epigenetic changes in lifestyle-related diseases are attracting attention as a possible mechanism of the memory effect, which was first described in large clinical trials of diabetes. The landmark Diabetes Control and Complications Trial (DCCT) and the Epidemiology of Diabetes Intervention and Complications (EDIC) study have shown that intensive glycemic control at the early stage of type 1 diabetes delays the progression of microvascular complications compared to conventional therapy despite similar mean hemoglobin A1c (HbA1c) levels at the later stage [2]. Another study has reported similar long-term benefits of intensive glycemic control in patients with type 2 diabetes [2, 3]. Furthermore, these memory effects in diabetes have been recognized in hypertension and atherosclerosis. In hypertension, animal models have demonstrated that transient administration of high-dose renin-angiotensin system (RAS) blockers or high-salt intake results in a sustained blood pressure reduction or an increase in blood pressure after cessation of the treatment, respectively [4, 5]. Such memory effects mediated by RAS are memorized in the kidney, especially in the kidney vasculature. The memory effect of RAS blockade may be feasible in humans [6, 7].

Recent studies using DCCT/EDIC cohorts have indicated that altered DNA methylation in blood cells, especially myeloid cells and hematopoietic stem cells, is significantly associated with complications of diabetes, including retinopathy and nephropathy [8, 9]; that is, DNA methylation changes in blood cells may denote the memory effect in diabetes for a long period. Targeting epigenetic changes is expected to be a new approach to treat chronic kidney disease that is progressively deteriorating due to its sustained efficacy.

This review summarizes novel strategies of treatment for kidney diseases, focusing on epigenetic alterations, especially their utility as markers and potential therapeutic targets.

## Epigenetic changes in glomerular podocytes

Podocytes form a slit membrane, which is a glomerular filtration barrier. Decreased expression of podocyte genes, which is required for renal integrity, causes disruption of the slit membrane, proteinuria and ultimately glomerulosclerosis [10, 11]. Epigenetic factors in podocytes control gene expression and activity in response to environmental changes.

We found that transient induction of transcription factor Kruppel-like factor 4 (KLF4) in podocytes causes a sustained decrease in albuminuria in a murine model of glomerulosclerosis [12]. Based on the unique characteristics of KLF4, which is one of the Yamanaka factors that induce iPS cells and has been reported to be involved in epigenetic remodeling at the reprogramming stage [13, 14], we performed a comprehensive methylome using KLF4-overexpressing podocytes. KLF4 mediates gene-specific DNA methylation in podocytes, but the gene-specific mechanism remains to be fully elucidated. Decreased KLF4 expression in podocytes causes increased DNA methyltransferase 1 (DNMT1) binding but causes decreased acetylated H3K9 in the nephrin promoter region, leading to a decrease in nephrin expression. Nephrin is an essential molecule that forms a slit membrane; therefore, decreased nephrin expression induces disruption of the slit membrane and proteinuria [12, 15]. Activated RAS in CKD causes decreased expression of podocyte KLF4, suggesting that podocyte KLF4 may contribute to a sustained effect of RAS blockade via a reset of epigenetic alterations in part [15, 16]. Future studies are necessary to clarify the extent to which epigenetic changes are triggered and which drug can rewrite the epigenome. Subsequently, the importance of KLF4 in podocytes in renal integrity has been reported by other groups. KLF4 negatively regulates STAT3-induced glomerular epithelial cell proliferation [17] and maintains parietal epithelial cell quiescence in the kidney [18]. KLF4 also contributes to the homeostasis of tubular epithelial cells and macrophages in the kidney [19, 20]. These results suggest that restoration of KLF4 expression may be a promising target for kidney disease.

A series of reports on epigenetic changes in podocytes has revealed the feasibility of the podocyte epigenome for therapeutic targets as well as disease-associated markers. In particular, we focused on DNA methylation changes because they are more stable than other epigenetic modifications and may contribute to sustained changes in gene expression. Methylation of cytosine in CpG islands, which are often found in or around the promoter region, usually causes repression of transcription. DNA methyltransferases mediate cytosine methylation using *S*-adenosyl-*l*-methionine as a methyl donor. Although the mechanism of DNA methylation in nondividing cells remains unclear, we reported that both DNMT1 and DNMT3B have a coordinated role in podocyte DNA methylation [21]. In neurons, which are also nondividing cells, it has been reported that both DNMT1 and DNMT3A play important roles in the plasticity of brain function [22]. Recently, it has been reported that activation of the KDM6A-KLF10 positive feedback loop in hyperglycemic states contributes to podocyte dysfunction through decreased nephrin expression by direct binding of KLF10 to the gene promoter together with the recruitment of DNMT1 [23]. Zhang et al. showed that podocyte DNMT1 may be a promising target for the treatment of diabetic nephropathy. Moreover, 5-azacytidine, a DNA methylation inhibitor, recovers nephrin expression and morphological changes in diabetic podocytes, leading to a reduction in albuminuria [24]. In addition, 5-aza-2ʹ-deoxycytidine, also an inhibitor of DNA methyltransferase, alleviates podocyte damage through the restoration of suppressed regulator of calcineurin 1 (RCAN1) expression in cultured podocytes [25]. Elevated 10-11-translocation 2 (Tet2), a DNA demethylation enzyme, is protective against podocytes [26]. These results suggest that reset or restoration of podocyte DNA methylation status, generally increased DNA methylation in disease states, may be a promising target for CKD.

## Role of DNA damage repair in the development of epigenetic changes

In proliferative cells, epigenetic changes are frequently caused by DNA replication errors at cell division [27]. The kidney consists of various types of cells with slowly proliferative or non-proliferative features; therefore, DNA damage/repair stress is expected to have a pivotal role in epigenetic alterations. DNA damage is caused by exogenous stress, such as ultraviolet radiation and chemicals, as well as endogenous stress, such as reactive oxygen species, stress hormones, DNA replication errors, spontaneous reactions and mechanical stress. When damaged DNA is repaired, epigenetic marks are also reconstituted. The DNA methyltransferase DNMT1 localizes to sites of DNA repair where it colocalizes with phosphorylated histone H2AX (γH2AX) to silence the repaired gene. Many chromatin modifiers regulate the assembly of repair machinery, lesion removal and restoration of the original chromatin state, which may leave epigenetic changes [28, 29]. Occasionally, some errors occur in the epigenetic repair process, and the accumulation of altered epigenetic marks causes aberrant gene expression [28]. However, the association of DNA damage repair with aberrant epigenetic states has not been adequately clarified, especially under in vivo conditions.

In dividing cells, 10–50 DNA double-strand breaks (DSBs) per day have been estimated to occur [30, 31]. A large amount of DSB stress may exist around podocytes, which are nondividing cells, but the number of DSBs induced in podocytes per day is not clear. Few DNA DSB sites have been observed in the kidneys of healthy young mice, whereas genomic and mitochondrial DNA damage sites are increased in podocytes under high-glucose conditions, suggesting that the DNA repair mechanism may be of great importance in podocytes [21, 32]. Because podocytes are not regenerated or replenished at baseline, the number of podocytes decreases with age [10, 33]. Therefore, the accumulation of podocyte DNA damage may contribute to podocyte loss and renal aging.

**Fig. 1:**
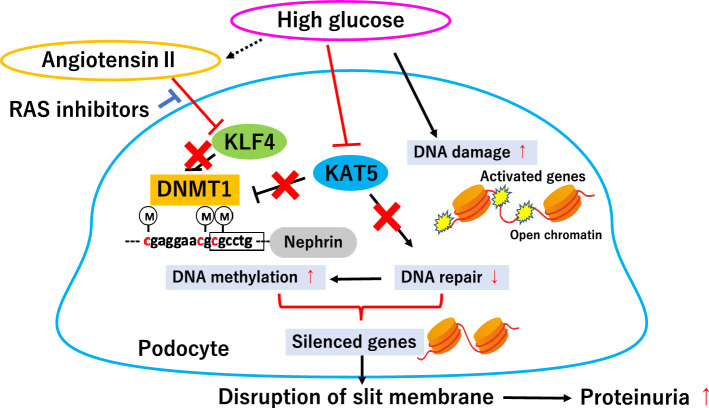
DNA damage repair and DNA methylation changes in damaged podocytes. An upregulated renin angiotensin system (RAS) induces a decrease in KLF4 expression in podocytes, leading to suppression of nephrin expression through increased DNMT1 binding and DNA methylation of the nephrin promoter. KLF4 may contribute to the sustained decrease in proteinuria induced by RAS inhibitors. High glucose causes decreased KAT5 expression in podocytes, and both increased DNA damage induction and decreased KAT5 expression promote DNA damage and increased DNA methylation in activated genes, which silence the expression of genes essential for podocyte function and cause disruption of the slit membrane and proteinuria. *KLF4* Kruppel-like factor 4, *KAT5* lysine acetyltransferase 5, *DNMT1* DNA methyltransferase 1

Lysine acetyltransferase 5 (KAT5) is a member of the MYST family of histone acetyltransferases and activates ATM and DNA-PKcs, which is an important factor for nonhomologous end-joining (NHEJ) repair. Acetylation of γH2AX by KAT5 induces the initiation of ubiquitination of γH2AX, which promotes the release of γH2AX from chromatin following DSBs [34]. Recently, we demonstrated that the DNA DSB repair factor KAT5, which acts coordinately with KLF4 [35], is indispensable for maintaining healthy podocytes and that loss of KAT5 in podocytes causes massive proteinuria and glomerulosclerosis with increased DNA damage sites and DNA methylation in podocytes. Increased DNA methylation in podocyte-specific genes, such as nephrin, causes silenced promoter activity and decreased expression. Furthermore, KAT5 expression in podocytes is reduced in both mouse models and humans with diabetic nephropathy, leading to attenuated DNA repair in podocytes with increased DNA damage induction due to high-glucose conditions [21]. These results indicate that metabolic changes, such as diabetes, affect the environment of DNA damage repair, which is associated with epigenetic changes. Figure 1 summarizes DNA damage repair and DNA methylation changes in damaged podocytes.

**Fig. 2:**
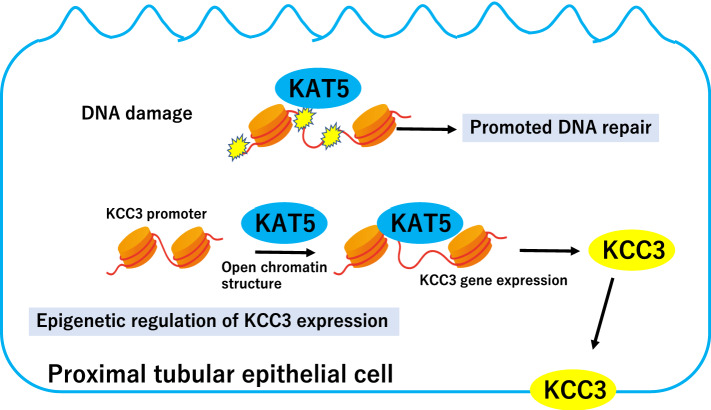
Roles of KAT5 in damaged proximal tubular epithelial cells. DNA repair factor KAT5 plays a multitasking role, not only in DNA repair but also in epigenetic regulation of KCC3 expression through the restoration of the original chromatin state, which could affect the remote hemodynamic changes. *KCC3* K-Cl cotransporter 3

The DNA repair system plays a central role in recovery and longevity in proximal tubular (PT) cells following AKI [36]. DNA repair factor KAT5 in PT cells acts differently from that in podocytes. PT cell-specific KAT5 knockout mice present mild tubular damage without elevated serum creatinine, whereas podocyte-specific KAT5 knockout mice show massive albuminuria and renal failure [21, 37]. It has been demonstrated that KAT5 plays an important role both in promoting DNA repair and in attenuating tubuloglomerular feedback (TGF) in an ischemic reperfusion (IR) induced-acute kidney injury (AKI) model in mice. Furthermore, K-Cl cotransporter 3 (KCC3) expression is decreased in damaged proximal tubular cells, resulting in increased chloride concentration in the macula densa, which may be involved in accelerated TGF following IR. It has been found that KAT5 induces KCC3 expression by maintaining chromatin accessibility and binding to the KCC3 promoter [37]. These results suggest the following important aspect of a DNA repair factor: it plays a multitasking role not only in the DNA repair of damaged cells but also in the regulation of remote hemodynamic changes, which may affect organ function (Fig. 2). In addition, genome-wide transcriptomic analysis of nephronophthisis PT cells with DNA repair factor deficiency has revealed dysregulated pathways in anion transport [38, 39], indicating that DNA repair factors in PT cells may be involved in electrolyte regulation as well as DNA repair.

Together with recent reports of the cell type-specific expression of DNA repair factors in single-cell RNA-seq in the kidney [40, 41], these results indicate that DNA repair factors or pathways may become organ- and cell-specific therapeutic targets. Further study is necessary to determine the dominant factors in the DNA repair pathway in various cell types of the kidney, which may develop a novel therapeutic target for disease therapy.

## Potential of DNA damage and DNA methylation as disease markers

Using kidney biopsy samples, DNA methylation of 5mC and DNA DSBs can be evaluated by immunostaining using antibodies against 5mC and γH2AX, respectively. In addition, quantitative assessment of DNA DSBs can be performed using a previously described long-distance PCR method [42]. We have previously reported the association of glomerular DNA methylation with glomerular DNA DSB levels in IgA nephropathy, which is correlated with eGFR decline per year [43].

Examination of urine samples is easy and less invasive than kidney biopsy, and these samples contain extracellular vesicles, such as exosomes and various types of cells. Extracellular vesicles from urine have attracted significant attention as potential diagnostic biomarkers in renal diseases [44]. The number of urinary podocytes is useful to diagnose early glomerular diseases and to evaluate disease activity [45]. In addition, evaluation of gene expression in urine-derived cells may be valuable. Altered expression of epigenetic modifiers, such as DNMTs and TETs, can be assessed in whole urine-derived cells. We have previously shown that DNMT/AQP1 expression is correlated with the eGFR decline rate [46]. Recently, single-cell RNA-seq analysis using urine-derived cells as well as biopsy samples has been reported [47, 48]. In the near future, single-cell analysis can be performed more easily and conveniently, and it is expected that even more human data can be accumulated in various clinical settings.

**Fig. 3:**
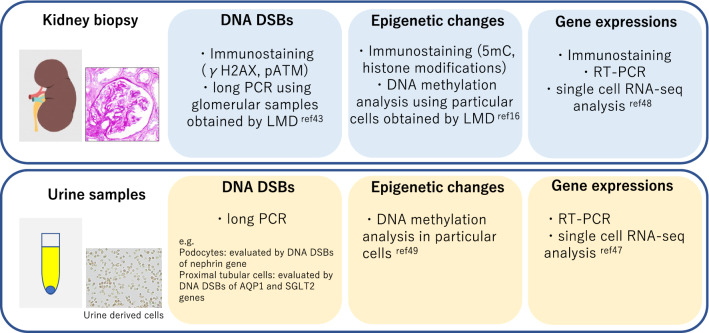
Evaluation of kidney biopsy and urine samples related to renal epigenetic changes. In humans, renal epigenetic changes can be evaluated using kidney biopsy or urine samples. This figure summarizes possible methods for detecting DNA double-strand break (DSB) epigenetic changes and altered gene expression in kidney biopsy and urine samples. *pATM* phosphorylated ataxia telangiectasia mutated, *LMD* laser microdissection, *AQP1* aquaporin 1, *SGLT2* sodium-glucose cotransporter 2

Epigenetic patterns in urine-derived cells are quite different among cell types, and the detection of epigenetic changes may be complicated. It has been indicated that the DNA methylation pattern of proximal tubule-specific loci in urine sediment is a potential marker of kidney function decline in diabetes [49]. Evaluation of DNA DSBs in urine-derived cells can be performed by the quantitative long-distance PCR method. Based on the assumption that DNA damage occurs in opened chromatin, DNA damage in the nephrin gene, which is specifically expressed in podocytes, may reflect the level of podocyte DNA damage [21, 46]. The evaluation of kidney biopsy and urine samples related to renal epigenetic changes is summarized in Fig. 3.

Recent epigenome-wide association studies (EWAS) have suggested that the DNA methylation status of saliva and blood cells is associated with kidney function [50, 51].

Of interest, some altered DNA methylation loci are common to the blood and kidney samples, but others are different. Although the mechanism underlying the development of DNA methylation changes in various cells associated with kidney function has not been elucidated, DNA methylation changes in these cells may be an interesting marker for renal function and prognosis.

## Conclusion

It is frequently asked whether an altered epigenome is a cause of disease or only a consequence of disease. Based on recent reports, an altered genome is, at least in part, a cause of disease. Approaches to modifying the renal epigenome seem to be feasible, but caution is merited owing to side effects of the treatment or the potential intergenerational effects. Focusing on transcription factors, such as KLF4, or DNA damage repair factors, such as KAT5, associated with epigenetic changes may be useful due to their cell-specific expression or action. We believe that a better understanding of the epigenetic alterations in the kidney and other organs or tissues related to kidney function will lead to new strategies against kidney aging and diseases in an aging society with an increasing CKD population.
